# Prevalence of vancomycin-variable *Enterococcus faecium* (VVE) among *vanA*-positive sterile site isolates and patient factors associated with VVE bacteremia

**DOI:** 10.1371/journal.pone.0193926

**Published:** 2018-03-22

**Authors:** Philipp Kohler, Alireza Eshaghi, Hyunjin C. Kim, Agron Plevneshi, Karen Green, Barbara M. Willey, Allison McGeer, Samir N. Patel, for the Toronto Invasive Bacterial Diseases Network (TIBDN)

**Affiliations:** 1 Microbiology Department, Mount Sinai Hospital, Toronto, Ontario, Canada; 2 Clinic for Infectious Diseases and Hospital Epidemiology, Cantonal Hospital St. Gallen, St. Gallen, Switzerland; 3 Public Health Ontario, Public Health Ontario Laboratory, Toronto, Ontario, Canada; 4 Department of Laboratory Medicine and Pathology, University of Toronto, Toronto, Ontario, Canada; Universidad Nacional de la Plata, ARGENTINA

## Abstract

Vancomycin-variable enterococci (VVE) are *vanA*-positive, vancomycin-susceptible enterococci with the ability to revert to a vancomycin-resistant phenotype on exposure to vancomycin. We sought to assess the prevalence of VVE and to determine clinical characteristics of patients infected with VVE. We prospectively collected *Enterococcus faecium* sterile site isolates from Toronto Invasive Bacterial Diseases Network hospitals from January 2015 to June 2016 and calculated VVE (defined as *vanA*-positive, vancomycin-susceptible isolates) prevalence among *vanA*-containing isolates. We performed chart reviews of VVE and vancomycin-resistant *E*. *faecium* (VRE) bacteremias identified from January 2012 to June 2016, and on a random sample of patients with bacteremia due to *vanA/vanB*-negative, vancomycin-susceptible enterococci (VSE) from January 2015 to June 2016. Clinical characteristics were compared and factors associated with mortality assessed. Because of the potential reversion from VVE to VRE, pulsed-field gel electrophoresis (PFGE) was performed for strains causing breakthrough bacteremia in order to identify relatedness among strains with different phenotypic resistance within the same patient. VVE comprised 47% (18/38) of *vanA*-positive isolates. The charts of 36 VRE, 25 VVE, and 79 VSE patients were reviewed. Central venous catheter associated bacteremia was more common in VVE (44%) and VRE patients (57%) than in VSE patients (28%) (P = 0.01). The Pitt bacteremia (OR 1.3, P = 0.002) and the Charlson score (OR 1.2, P = 0.008) were the only independent mortality predictors. PFGE of strains causing breakthrough bacteremia showed high within-patient clonality, irrespective of *vanA*-positivity or vancomycin-susceptibility. A substantial proportion of *vanA*-positive isolates are VVE and are therefore not detected with conventional selective culture methods. Bacteremia sources of patients with VVE are similar to those infected with VRE. We detected no association between VVE and 30-day mortality or breakthrough bacteremia.

## Introduction

*VanA*-positive enterococci with phenotypic susceptibility to vancomycin have been termed vancomycin-variable enterococci (VVE) and have been reported from Canada, South Korea, and Norway [1–5]. Molecular analyses have shown that VVE typically lack the *vanS* (sensor) and *vanR* (regulator) genes despite harbouring the *vanHAX* gene cassette [3, 6]. Because of their susceptibility to vancomycin, VVE escape the traditional detection methods for vancomycin-resistant enterococci (VRE), which might be associated with under-diagnosing and silent dissemination of VVE in healthcare facilities. Indeed, VVE have often been reported as clusters causing hospital outbreaks [3–5, 7]. Worryingly, both in *vivo* and *in vitro* data show that VVE have the ability to revert into vancomycin-resistant phenotypes upon vancomycin exposure [5, 6, 8].

The prevalence of VVE among *vanA*-positive enterococci has mostly been reported in single institution reports, but rarely on a multi-institutional or regional level [1, 3, 5, 6]. Despite the obvious challenges regarding diagnosis and infection control aspects of VVE, their clinical significance remains unknown. No data are available regarding risk profiles or outcomes of patients with VVE in comparison to VRE or *vanA*-negative vancomycin-susceptible enterococci (VSE). Whether patients infected with VVE can safely be treated with vancomycin or whether compounds active against VRE such as linezolid or daptomycin should be given, is not clear.

This study aimed to assess the prevalence of VVE among *vanA*-positive *Enterococcus faecium* sterile-site isolates in patients hospitalized in south-central Ontario, to compare the clinical characteristics of patients with VVE, VRE and VSE bacteremia, and to determine factors associated with 30-day mortality. Due to the ability of VVE to revert into VRE under treatment with vancomycin, we also aimed to describe clinical and microbiology details of patients with breakthrough *E*. *faecium* bacteremia with regard to the underlying resistance patterns.

## Materials and methods

### Setting

We prospectively collected all consecutive *E*. *faecium* (N = 372) isolates from sterile body sites (i.e. blood, ascites, pleural fluid) from microbiology laboratory members of the Toronto Invasive Bacterial Diseases Network (TIBDN) between January 2015 and June 2016 (prospective isolates). The TIBDN is a collaborative network of microbiology laboratories, infection-control practitioners, and public-health departments that performs population-based surveillance for infectious diseases in southern Ontario. In order to compare the clinical features of VRE, VSE and VVE infections, we also included *vanA*- or *vanB*-positive isolates from sterile site cultures (N = 37) which had been identified in the four TIBDN hospitals which screened all sterile site isolates of *E*. *faecium* for the presence of *vanA* and *vanB* from January 2012 to December 2014 (retrospective isolates).

### Microbiology

Vancomycin, ampicillin, linezolid and daptomycin susceptibility was tested by the individual licensed microbiology laboratories or by the Public Health Ontario Laboratories (PHOL) as per Clinical and Laboratory Standards Institute guidelines [9]. All isolates underwent polymerase chain reaction (PCR) testing for the presence of *vanA* and *vanB*, either at PHOL or at the local laboratories [3]. *VanA*- or *vanB*-positive isolates from local laboratories were confirmed at PHOL by multiplex real-time PCR. These isolates and those tested negative for *vanA* or *vanB* at PHOL underwent multiplex real-time PCR testing for the detection of regulatory genes *vanR* and *vanS* [3]. Isolates phenotypically susceptible to vancomycin were classified as VSE if they did not contain the *vanA* or *vanB* genes and as VVE if they were *vanA*-positive. Isolates that were phenotypically vancomycin-resistant and contained *vanA* were classified as VRE. *VanB*-containing isolates were classified as VRE irrespective of vancomycin-resistance.

Breakthrough bacteremia was defined as *E*. *faecium* bacteremia with a different susceptibility pattern to the original either during antibiotic therapy or within 30 days of its completion. If available, both original and breakthrough isolates of *E*. *faecium* underwent pulsed field gel electrophoresis (PFGE) to assess within-patient clonality of *E*. *faecium* isolates, using an established procedure [10]. After digestion with SmaI, the gel was run on CHEF-DR II instrument (Bio-Rad) and PFGE bands were analysed using BioNumerics (version 5.10) software. Isolates were defined as “same” if PFGE patterns were indistinguishable, as “related” if the Dice co-efficient indicated ≥75% similarity and “unrelated” if the Dice coefficient was <75% [3, 10].

### Patient data

We selected a random sample of prospectively identified VSE sterile site isolates, and performed chart reviews for all first episodes of VSE, VRE and VVE bacteremia from these isolates. Data on demographics, risk factors (hospital acquisition, days to diagnosis, intensive care stay, comorbidities and Charlson score [11], ward location on day of first culture, previous antibiotic treatment), infection source, data required for Pitt bacteremia score calculation [12], treatment (time to appropriate antibiotic treatment, surgery/intervention to control source of infection), and outcome (time to first negative blood culture, intensive care admission, in-hospital and 30-day mortality) were collected.

### Definitions

Infections were defined as hospital acquired if the specimen yielding *E*. *faecium* was obtained on or after day 3 of a hospital admission. The bacteremia source was determined based on attending physician diagnosis and chart review. Time to first negative blood culture was calculated for those who had at least one negative follow-up culture. Appropriateness of antibiotic treatment was defined as receiving at least one drug to which the isolate was phenotypically susceptible (for ampicillin and vancomycin) or to which the isolate was either susceptible or not tested (for linezolid, daptomycin, tigecycline). For patients with VVE, vancomycin was considered effective. *E*. *faecium* bacteremia was judged as being the cause of, contributing to, or being unrelated to 30-day mortality (i.e. after first positive culture).

### Analysis

VVE prevalence was calculated as percentage of all prospectively identified *vanA*-positive isolates (January 2015 to June 2016). Two different analyses were performed including prospective and retrospective isolates: i) comparison of patients with VRE, VVE, and VSE in terms of risks and outcomes, and ii) predictors of 30-day mortality. For each patient, only the first episode of bacteremia was included. Episodes of breakthrough bacteremia were described separately.

SAS software was used for all statistical analysis. A P-value <0.05 was considered statistically significant. Categorical variables were reported as frequencies and proportions, continuous variables as median with interquartile range (IQR). For dichotomous variables, Chi-square or Fisher-exact tests were used, as appropriate. For continuous variables, the Mann-Whitney-U or the Kruskal-Wallis tests were used. Univariable logistic regression was performed to estimate the association of patient-related factors and type of vancomycin resistance (i.e. VRE, VVE, and VSE) with 30-day mortality. Variables with a variance inflation factor <4 and a P-value ≤0.1 in univariable analysis were entered into a multivariable logistic regression model using automated stepwise variable selection.

### Ethics

This study involved hospital medical record review only, and all research ethics boards approved a waiver of consent for this study. Data were de-identified as soon as the medical records review process was complete. The study was approved the by research ethics boards/committees of the following institutional members of TIBDN: Baycrest Centre for Geriatric Care; Bridgepoint Active Healthcare; Halton Healthcare, Headwaters Healthcare Centre, Humber River Hospital, Joseph Brant Memorial Hospital, Lakeridge Health, Mackenzie Health, Markham Stouffville Hospital, Michael Garron Hospital, Mount Sinai Hospital, North York General Hospital, Orillia Soldiers Memorial Hospital, Public Health Ontario, Rouge Valley Health System, Royal Victoria Hospital, Southlake Regional Health Centre, St. Joseph’s Health Centre, St. Michael’s Hospital, Sunnybrook Health Sciences Centre, The Hospital for Sick Children, The Scarborough Hospital, Trillium Health Partners, University Health Network, William Osler Health System, Women’s College Hospital.

## Results

### VVE prevalence

A total of 372 episodes of illness associated with a sterile site isolate of *E*. *faecium* were identified by TIBDN hospitals between January 1, 2015 and June 30, 2016. Of these episodes, 350 (94%) were bacteremias, while 22 (6%) had non-blood sterile site isolates only. The *vanA* gene was detected in 38 (10.2%), of which 18 (47%) were VVE; two isolates (0.5%) were *vanB* positive. Among 37 retrospectively collected isolates from the four hospital sites, *vanA* was detected in 35 (11 VVE, 24 VRE), and *vanB* in 2; all were from blood cultures. All *vanA* or *vanB* containing isolates as well as a subset of isolates in which *vanA* or *vanB* were not detected (n = 184) were tested for the presence of *vanR/vanS*. Among *vanA*-positive isolates, VRE were more likely to carry *vanR/vanS* than VVE strains (86% *vs*. 31%, P<0.001) (Table 1). One of 184 *vanA/vanB*-negative isolates (0.5%) was also positive for *vanR/vanS*.

**Table 1:** Results of polymerase chain reaction for *vanR/vanS* and antimicrobial susceptibility for 77 sterile-site *Enterococcus faecium* isolates stratified by VRE and VVE.

	Total	VRE		VVE
		*vanA+*	*vanB+*	*vanA+*
Total	77 (100%)	44 (100%)	4 (100%)	29 (100%)
Prospective	40 (52%)	20 (46%)	2 (50%)	18 (62%)
Retrospective	37 (48%)	24 (54%)	2 (50%)	11 (38%)
*vanR/S*-positive	47 (61%)	38 (86%)	0 (0%)	9 (31%)
Resistant to				
Vancomycin	46 (60%)	44 (100%)	2 (50%)	0 (0%)
Ampicillin	77 (100%)	44 (100%)	4 (100%)	29 (100%)
Linezolid	1/76 (1%)	0 (0%)	0 (0%)	1/28^a^ (4%)
Daptomycin	0/76 (0%)	0 (0%)	0 (0%)	0/28^a^ (0%)

VRE, Vancomycin-resistant enterococci (i.e. *vanA*-positive and vancomycin-resistant OR *vanB*-positive); VVE, Vancomycin-variable enterococci (i.e. *vanA*-positive and vancomycin-susceptible)

^a^One VVE isolate was not stored and susceptibility testing for linezolid and daptomycin could not be performed

All 18 prospective VVE isolates were reported from only eight institutions, which contributed 260/372 (70%) of prospective *E*. *faecium* isolates (P = 0.006). The proportion of VVE among prospective *vanA*-positive isolates in all TIBDN hospitals was 57% (17/30) in 2015 and 13% (1/8) in 2016 (P = 0.06). In the four hospitals with all *vanA* containing isolates identified from 2012 to 2016, the percentage of VVE was 33% (1/3) in 2012, 25% (5/20) in 2013, 42% (5/12) in 2014, and 59% (10/17) in 2015, then decreased to 0% (0/3) in 2016 (P = 0.18). Among first patient isolates, resistance to linezolid was detected in 1 VVE, but not in VRE isolates. Resistance to daptomycin was not detected (Table 1). Resistance to ampicillin was observed in all VRE and VVE isolates. For 148/332 (45%) VSE isolates—mostly isolates which were not sent to PHOL for further analysis—ampicillin susceptibility was not reported. Among the 184 tested VSE isolates, 148 (80%) were resistant to ampicillin.

### Comparison of patients with VRE, VVE, and VSE bacteremia

Charts were available for review for patients with 36 VRE, 25 VVE and 79 VSE bacteremias. VRE and VVE patients tended to be younger than VSE patients (median age 61 and 62 *vs*. 64 years, P = 0.07) and they were more likely to be pre-treated with antibiotics (75% and 76% *vs*. 57%, P = 0.08). VRE and VVE patients were more likely to have a central line associated blood stream infection (CLABSI) as source of bacteremia (57% and 44% *vs*. 28% in VSE patients, P = 0.01) and less likely to suffer from intra-abdominal infections (IAI) compared to VSE patients (28% and 20% *vs*. 51%, p = 0.006). Time to adequate treatment was significantly different among the three groups (P<0.001). A majority (68%) of VVE patients received VRE-active (i.e. linezolid, daptomycin, or tygecycline) antibiotic therapy. Time to first negative blood culture as well as 30-day mortality were similar between groups (Table 2).

**Table 2:** Risk factors and outcome of patients with bacteremia caused by vancomycin-resistant (VRE), -variable (VVE), and -sensitive *Enterococcus faecium* (VSE), TIBDN hospitals, 2012–2016.

	VRE (n = 36)		VVE (n = 25)		VSE (n = 79)		P-value
Male sex	21	(58.3%)	15	(60.0%)	44	(55.7%)	0.89
Age, median (IQR)	61	(19)	62	(17)	64	(24)	0.07
Hospital-acquired	29	(80.6%)	22	(88.0%)	59	(74.7%)	0.35
Days to diagnosis (IQR)	22	(40)	19	(32)	14	(31)	0.23
Charlson score ≥6	10	(27.8%)	7	(28.0%)	17	(21.5%)	0.69
Diabetes	9	(25.0%)	7	(28.0%)	23	(29.1%)	0.9
Chronic kidney disease	12	(33.3%)	5	(20.0%)	14	(17.7%)	0.17
Cancer	19	(52.8%)	13	(52.0%)	36	(45.6%)	0.72
Lymphoma/Leukemia	15	(78.9%)	7	(53.8%)	19	(52.8%)	0.15
Solid organ	4	(21.1%)	6	(46.2%)	19	(52.8%)	0.07
Organ transplant	13	(36.1%)	5	(20.0%)	14	(17.7%)	0.09
Neutropenia	12	(33.3%)	6	(24.0%)	13	(16.5%)	0.13
Pitt bacteremia score ≥4, n = 129	8	(25.0%)	5	(22.7%)	21	(28.0%)	0.87
On antibiotic treatment^a^	27	(75.0%)	19	(76.0%)	45	(57.0%)	0.08
Vancomycin	2	(7.4%)	0	(0.0%)	3	(6.7%)	0.38
Source: Central venous catheter	20	(55.6%)	11	(44.0%)	22	(27.8%)	**0.01**
Source: Intraabdominal	10	(27.8%)	5	(20.0%)	40	(50.6%)	**0.006**
Candida co-infection	3	(8.3%)	2	(8.0%)	2	(2.5%)	0.31
Polymicrobial infection	13	(36.1%)	8	(32.0%)	27	(34.2%)	0.95
ICU admission, n = 103	5	(20.0%)	5	(29.4%)	13	(21.3%)	0.74
Surgery/procedure	3	(8.3%)	0	(0.0%)	10	(12.7%)	0.17
Hours to effective therapy, median (IQR)	55	(43)	30	(18)	26	(25)	**<0.001**
VRE-active^b^ treatment	31	(86.1%)	17	(68.0%)	5	(6.3%)	**<0.001**
Days to clearance (IQR), n = 100	4	(3)	4	(2)	3	(3)	0.15
30-day mortality	8	(22.2%)	11	(44.0%)	23	(29.1%)	0.18
Attributable^c^ to *E*. *faecium*	7	(87.5%)	10	(90.9%)	18	(78.3%)	0.86

IQR, Interquartile Range; ICU, Intensive Care Unit

^a^On day of first positive culture

^b^Linezolid, daptomycin, or tigecycline

^c^*E*. *faecium* being the cause or contributing to death

### Predictors of mortality

Factors associated with 30-day mortality in univariable analysis were the Pitt bacteremia (OR 1.3, 95% CI 1.1–1.5) and the Charlson score (OR 1.3, 95% CI 1.1–1.4). These variables, along with the type of vancomycin resistance (VVE *vs*. VSE: OR 1.9, 95% CI 0.8–4.8; VRE *vs*. VSE: OR 0.7, 95% CI 0.3–1.8) and intervention for source control (OR 0.2, 95% CI 0.0–1.4), were entered into multivariable analysis. After adjustment, Pitt bacteremia score (OR 1.3, 95% CI 1.1–1.5) and Charlson score (OR 1.2, 95% CI 1.1–1.5) were the only independent predictors of 30-day mortality (Table 3).

**Table 3:** Univariable and multivariable logistic regression analyses to assess 30-day mortality among patients with *E*. *faecium* bacteremia.

	Univariable analysis		Multivariable analysis	
	OR (95% CI)	P-value	OR (95% CI)	P-value
Male sex	1.8 (0.8–3.8)	0.14		
Age, per year	1.01 (0.99–1.03)	0.51		
Charlson, per point	1.2 (1.1–1.4)	**0.006**	1.2 (1.1–1.5)	**0.008**
Pitt bacteremia score, per point	1.3 (1.1–1.5)	**0.003**	1.3 (1.1–1.5)	**0.002**
Resistance type		0.40	non-significant	
VSE	Reference	-		
VVE	1.9 (0.8–4.8)	0.08		
VRE	0.7 (0.3–1.8)	0.14		
Source of infection		0.33		
Other	Reference	-		
Primary CLABSI	0.6 (0.2–1.5)	0.15		
Intra-abdominal	1.1 (0.4–2.7)	0.35		
Surgery/procedure	0.2 (0.0–1.4)	0.10	non-significant	
Polymicrobial infection	0.9 (0.4–2.0)	0.88		
VRE-active^a^ treatment	0.7 (0.3–1.4)	0.27		
Hours to effective therapy, per hour	1.00 (0.99–1.01)	0.67		

VRE, Vancomycin-resistant enterococci; VVE, Vancomycin-variable enterococci; VSE, Vancomycin-susceptible enterococci; CLABSI, Central Line Associated Blood Stream Infection; OR, Odds Ratio; CI, Confidence Interval

^a^Linezolid, daptomycin, or tigecycline

### Episodes of breakthrough bacteremia

Among nine patients with breakthrough *E*. *faecium* bacteremia, the second positive blood culture occurred from 1 to 34 days after the first culture. Only one (patient E) of nine patients presenting initially with VVE bacteremia developed VRE bacteremia while being treated with vancomycin. In-hospital mortality was 78% (7/9) for these cases; the *E*. *faecium* infection was judged as contributing to 3 deaths (C, D, and F) (Table 4).

**Table 4:** Characteristics and outcomes of patients with breakthrough bacteremia due to *Enterococcus faecium* with different antibiotic susceptibility.

#	Age, Sex	Underlying conditions	Isolate 1 (on day 1)		Isolate 2		Isolate 3		Outcome, Role of *E*. *faecium* in case of death
			Type	SOURCE Antibiotic	Type (PFGE^a^) Day	SOURCE Antibiotic	Type (PFGE^a^) Day	SOURCE Antibiotic	
A	62, M	Leukemia, Neutropenia	VSE	CLABSI Dapto, Linez	VRE (same) 1	CLABSI Dapto, Linez			Discharge day 59 -
B	73, F	Leukemia, Neutropenia	VSE	CLABSI Vanco	VRE (NA) 6	CLABSI Linez, Dapto			Discharge day 24 -
C	61, F	Leukemia, Neutropenia (Muc)	VSE	ENDOCARD Dapto	VSE^b^ (same) 8	ENDOCARD Linez			Death day 55 Contributed
D	20, F	Liver transplant, Renal dialysis	VSE	PRIMARY Vanco	VRE (different) 15	CLABSI Dapto			Death day 18 Contributed
E	56, M	Leukemia, Neutro-penia, DM 2	VVE	PRIMARY Vanco	VRE (NA) 6	PRIMARY Linez			Death day 20 Unrelated
F	66, M	Liver transplant, Dialysis, DM 2	VVE	IAI Linez, Dapto	VVE (same) 34	IAI Dapto, Linez	VVE (same) 43^c^	IAI Linezolid	Death day 101 Contributed
G	22, M	Leukemia, Neutropenia (Muc)	VRE	CLABSI Linez	VSE (NA) 4	CLABSI Linez			Death day 197 Unrelated
H	63, M	Leukemia, Neutropenia (Muc)	VRE	IAI Dapto	VSE (related^d^) 21	IAI Vanco	VRE (related^d^) 33	IAI Linezolid	Death day 57 Unrelated
I	63, M	Chondrosarcoma, DM 2	VRE	CAUTI Vanco, Linez	VVE (same) 30	CLABSI Linez,Vanco			Death day 154 Unrelated

VRE, Vancomycin-resistant enterococci (i.e. *vanA*-positive and vancomycin-resistant OR *vanB*-positive); VVE, Vancomycin-variable enterococci (i.e. *vanA*-positive and vancomycin-susceptible); VSE, Vancomycin-susceptible enterococci (i.e. *vanA*- and *vanB*-negative); Muc, Mucositis; DM 2, Diabetes mellitus type 2; NA, Not Available (for typing); CLABSI, Central/Arterial Line Associated Blood Stream Infection; IAI, Intraabdominal Infection; ENDOCARD, Endocarditis; PRIMARY, Primary blood stream infection; CAUTI, Catheter Associated Urinary Tract Infection; Vanco, Vancomycin; Dapto, Daptomycin; Linez, Linezolid

^a^Relatedness of isolate from this blood culture to previous blood culture(s) based on Pulsed Field Gel Electrophoresis (PFGE)

^b^Daptomycin-resistant

^c^Patient had another VVE isolate (Linezolid-resistant) on day 59 with the same PFGE pattern. Treatment was switched to tigecycline and daptomycin.

^d^Isolate 2 was one band different to isolate 1, and isolate 3 was three bands different than isolate 2.

Within-patient comparison of *E*. *faecium* strains by PFGE showed that among five of six patients, where PFGE was performed on all strains, breakthrough bacteremia was caused by the same pulsotype despite differences in underlying resistances patterns. Between-patient comparison showed eight different *E*. *faecium* pulsotypes among the nine patients.

## Discussion

In south-central Ontario, between January 2015 and June 2016, the prevalence of VVE was 47% among *vanA*-positive sterile site isolates. Almost ninety percent of VVE bacteremia cases were hospital acquired, and the clustering within hospitals suggests that VVE are nosocomial pathogens endemic to some, but not all hospitals. For VRE and VVE, the most common BSI source was a central line, whereas VSE bacteremia was most commonly associated with gastrointestinal infection.

In our geographic area, VVE comprise a relatively high proportion of *vanA* containing enterococci which in Canada are primarily nosocomial pathogens [13–15]. Transmission of the *vanA* gene cassette is largely due to horizontal gene transfer of plasmids containing Tn*1546*-like elements [9]. Because this also seems to be the case for VVE, and because the frequent genetic rearrangements mean that VVE can be either resistant or susceptible to vancomycin, we believe that control of VVE in our geographic area will be important if we wish to protect our hospitals and patients from VRE [7]. Institutions or regions wishing to prevent the development of endemic VRE within their hospitals should be alert to the risk of VVE strains, and should consider using PCR for *vanA/vanB* genes for screening to contain their spread as needed. PCR performed directly after isolate identification not only detects *vanA*-positive isolates irrespective of phenotypic vancomycin-resistance but also significantly reduces the turnaround time of resistance results [16].

It has previously been shown that bacteremia with VRE—in contrast to VSE—is more often associated with central venous catheters, whereas VSE bacteremia is more frequently seen among patients with gastrointestinal disease, suggesting differences in pathogenesis [17, 18]. In this regard, VVE is more similar to VRE, likely because they are hospital-acquired pathogens. Predictors of mortality identified in this study were markers of underlying comorbidities and severity of disease, whereas vancomycin-resistance and time to adequate treatment were not associated with increased mortality. This contrasts the results of two meta-analyses showing an increased mortality associated with VRE- as compared to VSE-bacteremia [19, 20]. The most likely explanation is that our study was underpowered to detect a clinically significant difference. The relatively high proportion of our VRE that were CLABSI as compared to the higher proportion of IAI that were due to VSE may also have contributed, as CLABSI is known to have a lower case fatality rate than other types of bacteremia [21]. Of note, there was a non-significant difference in 30-day mortality between patients with VRE (22%), VVE (44%), and VSE (29%), with an unadjusted OR of 1.9 for patients with VVE, although most VVE patients were treated with VRE-active antibiotics. These results are interesting in light of a previously discussed potential gain of fitness for VVE. It has been postulated that insertion of an IS*L3*-like element between the VanR binding site and the *vanHAX* promoter region is responsible for silencing of the *vanA*-gene in VVE strains, resulting in a functional fitness gain [5]. Although the effect was less pronounced after adjusting for underlying comorbidities, further clinical studies with larger sample sizes should evaluate this finding.

Linezolid resistance was uncommon among all tested *E*. *faecium* isolates. Only two isolates (both VVE) showed resistance to linezolid, one from a baseline sample and one from a patient with breakthrough bacteremia who received linezolid before detection of the resistant strain. No resistances against daptomycin were observed, except for one patient with breakthrough bacteremia being treated with daptomycin.

This study found that breakthrough *E*. *faecium* bacteremia is usually caused by the same bacterial strain, a finding which has been reported for patients with recurrent *E*. *faecium* bacteremia [22]. Leukemia, previously shown to be a risk factor for breakthrough and recurrent bacteremia, was present in six of nine patients [22–24]. The severe underlying comorbidities in these patients also explain the high case fatality rate of 78%, which was mostly unrelated to the bacteremia. Serum drug concentrations of antibiotic substances used were not determined in these patients, although low drug concentrations have also been discussed as a risk factor for breakthrough bacteremia [25]. VVE patients were not overrepresented among patients with breakthrough bacteremia. However, there were only eight VVE patients in our study who were treated with vancomycin, so that we cannot rule out the possibility that selection for vancomycin resistance is important in this setting.

The inclusion of retrospective isolates and the small sample size are both limitations of this study and prevented us from drawing definitive conclusions as to the clinical significance of VVE. Although there is evidence that VRE in North America are almost exclusively hospital acquired, hospitals in our population area do not screen by PCR for VRE [13–15]. We did not attempt to identify where VRE or VVE were acquired in patients who developed bacteremia, so that we do not have proof that the organisms are not community acquired. Another limitation is the lack of more detailed microbiologic comparison of VVE isolates (e.g. plasmid sequencing, whole genome sequencing), to document similarities and differences between strains. There was substantial variability in VVE prevalence in different hospitals within our population area, such that our data cannot be generalized to other hospitals or other geographic areas. VVE may also occur in strains of different genetic background, and by a variety of different molecular mechanisms, such that both virulence and the risk of selection for vancomycin resistance among VVE strains may differ. Our decision to classify all *vanB*-positive isolates as VRE (irrespective of vancomycin-resistance) can be debated. However, because we identified only two *vanB*-positive, vancomycin-susceptible isolates, their inclusion or exclusion does not impact our results.

## Conclusions

In summary, the prevalence of VVE is high among *vanA*-positive *E*. *faecium* isolates in hospitals in south-central Ontario, Canada. Our data suggest that VVE are predominantly nosocomial pathogens, with epidemiology similar to VRE. Prior data on selection for vancomycin resistance in VVE, and our non-significant findings of an increased mortality for VVE patients suggest that patients with invasive VVE infections should be managed as if their infections were due to VRE until additional data are available.
